# High taxonomic level fingerprint of the human intestinal microbiota by Ligase Detection Reaction - Universal Array approach

**DOI:** 10.1186/1471-2180-10-116

**Published:** 2010-04-19

**Authors:** Marco Candela, Clarissa Consolandi, Marco Severgnini, Elena Biagi, Bianca Castiglioni, Beatrice Vitali, Gianluca De Bellis, Patrizia Brigidi

**Affiliations:** 1 Department of Pharmaceutical Science, University of Bologna, Italy; 2 Institute of Biomedical Technologies - Italian National Research Council, Milan, Italy; 3 Institute of Agricultural Biology and Biotechnology - Italian National Research Council, Milan, Italy

## Abstract

**Background:**

Affecting the core functional microbiome, peculiar high level taxonomic unbalances of the human intestinal microbiota have been recently associated with specific diseases, such as obesity, inflammatory bowel diseases, and intestinal inflammation.

**Results:**

In order to specifically monitor microbiota unbalances that impact human physiology, here we develop and validate an original DNA-microarray (HTF-Microbi.Array) for the high taxonomic level fingerprint of the human intestinal microbiota. Based on the Ligase Detection Reaction-Universal Array (LDR-UA) approach, the HTF-Microbi.Array enables specific detection and approximate relative quantification of 16S rRNAs from 30 phylogenetically related groups of the human intestinal microbiota. The HTF-Microbi.Array was used in a pilot study of the faecal microbiota of eight young adults. Cluster analysis revealed the good reproducibility of the high level taxonomic microbiota fingerprint obtained for each of the subject.

**Conclusion:**

The HTF-Microbi.Array is a fast and sensitive tool for the high taxonomic level fingerprint of the human intestinal microbiota in terms of presence/absence of the principal groups. Moreover, analysis of the relative fluorescence intensity for each probe pair of our LDR-UA platform can provide estimation of the relative abundance of the microbial target groups within each samples. Focusing the phylogenetic resolution at division, order and cluster levels, the HTF-Microbi.Array is blind with respect to the inter-individual variability at the species level.

## Background

Human beings have been recently reconsidered as superorganisms in co-evolution with an immense microbial community living in the gastrointestinal tract (GIT), the human intestinal microbiota [1,2]. Providing important metabolic functions that we have not evolved by our own [3], the intestinal microbiota has a fundamental role for the human health and well being [4,5]. Several of our physiological features, such as nutrient processing, maturation of the immune system, pathogen resistance, and development of the intestinal architecture, strictly depend on the mutualistic symbiotic relationship with the intestinal microbiota [6]. On the basis of its global impact on human physiology, the intestinal microbiota has been considered an essential organ of the human body [7].

The composition of the adult intestinal microbiota has been determined in three large scale 16S rRNA sequences surveys [7-11]. The phylogenetic analysis of a total of 45,000 bacterial 16S rRNA data from 139 adults revealed that, at the phylum level, only a small fraction of the known bacterial diversity is represented in our GIT. The vast majority of bacteria in the human intestinal microbiota (>99%) belongs to six bacterial phyla: *Firmicutes, Bacteroidetes, Actinobacteria, Proteobacteria, Fusobacteria and Verrucomicrobia*. The two dominant divisions are *Firmicutes *and *Bacteroidetes*, which represent together up to 90% of the total microbiota, with a relative abundance of 65% and 25%, respectively. *Actinobacteria*, *Proteobacteria, Verrucomicrobia *and *Fusobacteria *are the subdominants phyla with a relative abundance up to 5, 8, 2 and 1%, respectively. On the contrary, at lower taxonomic levels, we assist to a real explosion of the bacterial diversity in the human GIT. At least 1,800 genera [≥ 90% of sequence identity (ID)] and 16,000 phylotypes at the species level (≥ 97% ID) have been identified until now, predicting even a greater diversity at the species level [8]. Since 70% of these phylotypes are subject-specific, and no phylotype is present at more than 0.5% abundance in all subjects [12], the intestinal microbiota of each individual has been shown to consist in a subject specific complement of hundreds of genera and thousands of species. However, the large degree of functional redundancy between species and genera allowed identifying a core microbiome at the gene level which is shared between all individuals [12]. Coding for genes involved in important metabolic functions, this core functional microbiome is fundamental to support the mutualistic symbiotic relationship with the human host.

Recently, 16S rRNA sequences studies have been carried out with the attempt to describe disease-associated unbalances of the human intestinal microbiota. Even though species variability was associated with inter-individual variability, phylum-level changes of the intestinal microbiota were associated with specific diseases. In particular, obesity was characterized by a higher proportion of *Firmicutes *and *Actinobacteria *with respect to *Bacteroidetes *and an overall reduced bacterial diversity [12,13]. Differently, inflammatory bowel diseases (IBD) were characterized by a marked reduction of bacterial diversity in the *Clostridium *cluster IV and XIVa belonging to *Firmicutes*, a decline in *Bacteroidetes *biodiversity, and a correspondent increase in *Proteobacteria *and *Bacillus *[14,15]. Analogously, intestinal inflammation has been generally related with a marked increase in *Enterobacteriaceae *and a correspondent decrease in members of the resident colonic bacteria [16,17]. In the light of these findings, it has been recently hypothesized that these high level taxonomic unbalances of the human intestinal microbiota can cause deviations from the core functional microbiome with a final impact on the host physiological state [12,18,19].

Since more than 75% of the phylotypes detected in the human GIT does not correspond to cultured species [20], phylogenetic DNA-microarrays have been recognized as a valuable tool for a high-throughput, quantitative and systematic analysis of the human intestinal microbiota [21]. Recently, three different small ribosomal subunit RNA (SSU rRNA) based high-density phylogenetic microarrays for studying the human microbiota have been developed [22-24]. Targeting thousands bacterial phylo-types, these DNA-microarrays have been successfully applied in studies for the deep phylogenetic characterization of the human intestinal microbiota.

In order to specifically monitor the microbiota unbalances that impact on human physiology independently of the inter-individual variability, here we developed an original DNA-microarray for the high taxonomic level fingerprint of the human intestinal microbiota, called HTF-Microbi.Array (High Taxonomic Fingerprint Microbiota Array). The relatively low number of targets allowed implementing the Ligase Detection Reaction (LDR) technology [25,26] for the development of the HTF-Microbi.Array. This enzymatic *in vitro *reaction, based on the discriminative properties of the DNA ligation enzyme, requires the design of a pair of two adjacent oligonucleotides specific for each target sequence: a probe specific for the variation (called "Discriminating Probe", or DS) which carries a 5'-fluorescent label, and a second probe, named "Common Probe" (or CP), starting one base 3'-downstream of the DS that carries a 5'-phosphate group and a unique sequence named cZipCode at its 3'-end. The oligonucleotide probe pairs and a thermostable DNA ligase are used in a LDR reaction with previously PCR-amplified DNA fragments. This reaction is cycled to increase product yield. The LDR products, obtained only in presence of a perfectly matching template by action of the DNA ligase, are addressed to a precise location onto a Universal Array (UA), where a set of artificial sequences, called Zip-codes are arranged. These products carry both the fluorescent label and a unique cZipCode sequence and can be detected by laser scanning and identified according to their location within the array. The LDR approach is a highly specific and sensitive assay for detecting single nucleotide variations; thus, differences of a single base along the 16S rRNA gene can be employed to distinguish among different microbial lineages. The HTF-Microbi.Array was successfully tested in a pilot study for the characterization of the faecal microbiota of eight healthy young adults.

## Results

### Target selection and probe design

**Figure 1:**
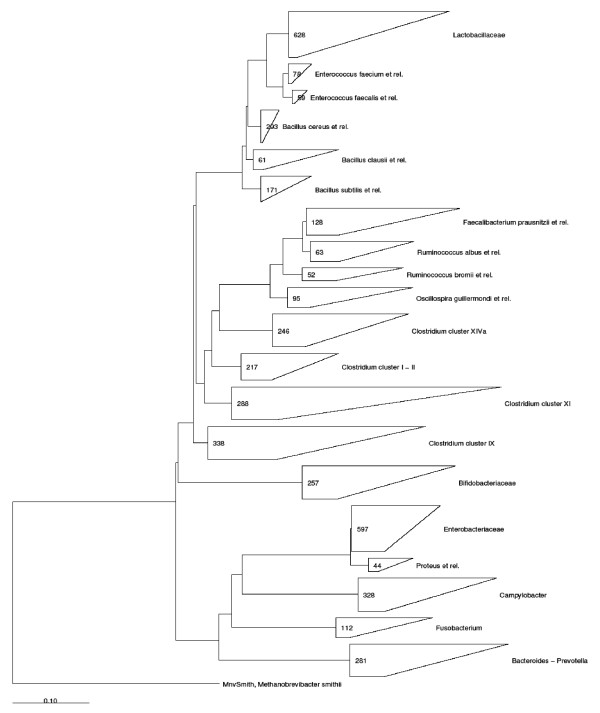
**SSU rRNA based phylogenetic tree of the 16S rRNA sequences of the HTF-Microbi.Array positive set**. For each node we report the number of sequences used from our ARB 16S rRNA sequence database. The triangles dimension is proportional to the number of sequences clustered together. The phylogenetic tree was obtained by using the neighbour-joining algorithm for the sequence alignment in ARB software.

The rational selection of the HTF-Microbi.Array targets was carried out using a phylogenetic approach. To this aim we implemented the 16S rRNA database of the ARB Project (release February, 2005) with the 16S rRNA gene database of the RDP available at the time and a phylogenetic tree was constructed. Based on the tree nodes, 30 phylogenetical groups of the human intestinal microbiota were rationally selected as the target group for the HTF-Microbi.Array (Additional file 1). In Fig. 1 we report the phylogenetic tree of the 16S rRNA sequences of the HTF-Microbi.Array positive set. The selected groups belonged to different phylogenetic levels (species, genus, family, cluster, or group of species indicated by the warding "*et rel*."). The entire list of the array targets is represented in Table 1. For part of the division *Firmicutes*, the target selection was carried out based on the classification proposed by Collins *et al. *[27]. *Clostridium *cluster I and II, *Clostridium *cluster IX, *Clostridium *cluster XI, and *Clostridium *cluster XIVa were selected. For the *Clostridium *cluster IV, four subgroups of species were defined: *Ruminococcus albus et rel., Ruminococcus bromii et rel., Faecalibacterium prausnitzii et rel.*, and *Oscillospira guillermondii et rel*. Within the *Firmicutes *division, the family *Lactobacillaceae*, and the groups *Bacillus clausii et rel., Bacillus subtilis et rel., Bacillus cereus et rel., Enterococcus faecalis et rel.*, and *Enterococcus faecium et rel. *were also selected. Other selected groups were the *Bacteroides/Prevotella *cluster (division *Bacteroidates*), the family *Bifidobacteriaceae *(division *Actinobacteria*), the family *Enterobacteriaceae *and the genus *Campylobacter *(division *Proteobacteria*). For clusters or families, relevant species, genera or subgroups of species were selected to design "sub-probes". The genus *Veillonella *was selected for *Clostridium *cluster IX, the species *Eubacterium rectale *for *Clostridium *cluster XIVa, *Clostridium difficile *for *Clostridium *cluster XI, and *Clostridium perfringens *for *Clostridium *cluster I and II. The group *Bifidobacterium longum et rel*. was chosen for the family *Bifidobacteriaceae*, and the genera *Yersinia *and *Proteus *for the *Enterobacteriaceae*. Based on an original phylogenetic design, the entire probe set of the HTF-Microbi.Array cover up to 95% of the bacterial groups belonging to the human intestinal microbiota [28].

**Table 1:** Probe set of the HTF-Microbi.Array.

PROBE	N.	TAXONOMIC LEVEL	CLUSTER	ORDER	DIVISION	ECO	H.G. AB %
*Bacteroides/Prevotella*	16	Cluster	*Bacteroides/Prevotella*	*Bacteroidales*	*Bacteroidetes*	M	20

*Ruminococcus bromii*	38	Sub cluster	Cl IV	*Clostridiales*	*Firmicutes*	M	
*Ruminococcus albus*	39	Sub cluster	Cl IV	*Clostridiales*	*Firmicutes*	M	
*Faecalibacterium prausnitzii*	40	Sub cluster	Cl IV	*Clostridiales*	*Firmicutes*	M	
*Oscillospira guillermondii*	41	Sub cluster	Cl IV	*Clostridiales*	*Firmicutes*	M	65
	
*Clostridium *IX	37	Cluster	Cl IX	*Clostridiales*	*Firmicutes*	M	
*Veilonella*	20	Species (*et rel*)	Cl IX	*Clostridiales*	*Firmicutes*	M	
	
*Clostridium *XIVa	22	Cluster	Cl XIVa	*Clostridiales*	*Firmicutes*	M	
*Eubacterium rectale*	19	Species (*et rel*)	Cl XIVa	*Clostridiales*	*Firmicutes*	M	

*Bifidobacteriaceae*	25B	Family	*Bifidobacterium*	*Bifidobacteriales*	*Actinobacteria*	M	5
*B. longum*	3	Species (*et rel*)	*Bifidobacterium*	*Bifidobacteriales*	*Actinobacteria*	M	

*Lactobacillaceae*	21B	Family	*Lactobacillaceae*	*Lactobacillales*	*Firmicutes*	M	
*L. plantarum*	33	Species (*et rel*)	*Lactobacillaceae*	*Lactobacillales*	*Firmicutes*	M	<1
*L. casei*	12	Species (*et rel*)	*Lactobacillaceae*	*Lactobacillales*	*Firmicutes*	M	
*L. salivarius*	14	Species (*et rel*)	*Lactobacillaceae*	*Lactobacillales*	*Firmicutes*	M	

*Bacillus clausii*	32	Species (*et rel*)	*Bacillaceae*	*Bacillales*	*Firmicutes*	M	<1

*Bacillus subtilis*	8	Species (*et rel*)	*Bacillaceae*	*Bacillales*	*Firmicutes*	M	<1

*Fusobacterium*	15	Genus	*Fusobacteriaceae*	*Fusobacteria*	*Fusobacteria*	M	<0.5

*Cyanobacteria*	42	Family	*Cyanobacteria*	*Cyanobacteria*	*Cyanobacteria*	M	<0.1

*Clostridium *XI	36	Cluster	Cl XI	*Clostridiales*	*Firmicutes*	O	0
*Clostridium difficile*	18	Species (*et rel*)	Cl XI	*Clostridiales*	*Firmicutes*	O	

*Clostridium *I and II	35	Cluster	Cl I and II	*Clostridiales*	*Firmicutes*	O	0
*Clostridium perfringens*	17	Species (*et rel*)	Cl I and II	*Clostridiales*	*Firmicutes*	O	

*Enterococcus faecalis*	9	Species (*et rel*)	*Enterococcales*	*Lactobacillales*	*Firmicutes*	O	<1

*Enterococcus faecium*	10	Species (*et rel*)	*Enterococcales*	*Lactobacillales*	*Firmicutes*	O	<1

*Bacillus cereus*	7	Species (*et rel*)	*Bacillaceae*	*Bacillales*	*Firmicutes*	P	0

*Enterobacteriaceae*	23B	Family	*Enterobacteraceae*	*Enterobacterales*	*Proteobacteria*	O/P	<8
*Yersinia enterocolitica*	4	Species (*et rel*)	*Enterobacteraceae*	*Enterobacterales*	*Proteobacteria*	O/P	0
*Proteus*	5	Genus	*Enterobacteraceae*	*Enterobacterales*	*Proteobacteria*	O/P	0

*Campylobacter*	6	Genus	*Campylobacteraceae*	*Campylobacterales*	*Proteobacteria*	P	0

For each probe is indicated the spot number, the phylogenetic level, the phylogeny of the target group, the ecology in the gastrointestinal ecosystem [mutualistic (M), opportunistic (O), pathogen (P)]. The relative abundance in a healthy gut ecosystem of the principal microbial groups is also indicated.

Specificity and coverage of each candidate probe was assessed by using the tool Probe Match of the RDP database. The probe pairs selected for the HTF-Microbi.Array were required to perfectly match the sequences of the positive set and to possess at least a mismatch at the 3' end of the discriminating probe respect to the entire negative set. The designed probes pairs had an average melting temperature (Tm) of 67.8 ± 0.9°C (n = 60) and an average length of 35.6 ± 4.9 nucleotides. Sixteen out of the 30 probe pairs were characterized by having no degenerated bases, whereas only one probe pair (i.e. the one for *Clostridium *cluster I and II) had 4 and 3 ambiguous bases on DS and CP, respectively (Additional file 2).

### Validation of the HTF-Microbi.Array

#### LDR probe pair specificity

The specificity of the designed LDR probe pairs was tested by using 16S rRNA PCR amplicons from 28 microorganisms members of the human intestinal microbiota. Amplicons were prepared by amplification of genomic DNA extracted from DSMZ cultures or genomic DNA from ATCC collection. Proving the specificity of the HTF-Microbi.Array all the 16S rRNA amplicons were properly recognized in separate LDR hybridization reactions with the entire probe set of the array. Two replicated independent LDR-UA experiments were performed with an optimal reproducibility (Additional file 3). For each of the 16S rRNA template only group-specific spots, and spots corresponding to the hybridization controls showed positive signals (*P *< 0.01) (Table 2). As a negative control, we performed two independent PCR-LDR-UA experiments using double distilled water, instead of genomic DNA, as sample. As expected, no positive signal was detected. The ratio between the signal intensities of the specific probes and the blank intensity (SNR_s_) averaged 206.9 ± 185.7, whereas the ratio between all the other probes and the blank intensity (SNR_ns_) averaged 2.1 ± 1.4. Therefore, the ratio between specific and non-specific probes resulted more than 100 fold on average.

**Table 2:** Specificity test.

DNA Target	Positive signal	SNR other	SNR spec	p-valus spec
*B. fragilis *ATCC25285	*Bacterodes/Prevotella*	0.85	30.81	9.35E-05
		
		0.53	21.45	7.39E-04

*B. thetaiotaomicrom *ATCC29143	*Bacterodes/Prevotella*	0.45	61.44	2.56E-04
		
		1.66	347.24	9.10E-06

*L. gasseri *DSM20243	*Lactobacillaceae*	0.30	5.58	4.98E-03
		
		1.56	20.59	6.58E-03

*P. melaninogenica *ATCC25845	*Bacterodes/Prevotella*	1.54	480.24	6.02E-08
		
		0.90	266.63	3.74E-09

*B. subtilis *DSM704	*Bacillus subtilis*	7.93	637.39	1.56E-09
		
		5.62	350.10	1.47E-05

*E. coli *ATCC11105	*Enterobacteriaceae*	3.27	555.04	8.65E-08
		
		2.59	222.39	4.50E-07

*P. mirabilis *DSM4479	*Proteus, Enterobacteriaceae*	2.42	703.22	7.74E-09
		
		2.03	497.10	1.97E-09

*B. bifidum *DSM20456	*Bifidobacteriaceae*	2.67	289.39	4.78E-11
		
		2.23	407.10	2.40E-08

*L. casei *DSM20011	*Lactobacillaceae, L. casei*	2.59	125.13	1.01E-04
		
		2.26	134.78	5.92E-04

*Y. enterocolitica *(faecal isolate)	*Yersinia enterocolitica, Enterobacteriaceae*	1.53	231.33	1.01E-05
		
		2.89	340.20	1.61E-06

*B. cereus *DSM31	*Bacillus cereus*	2.83	193.85	1.53E-06
		
		2.49	196.82	4.16E-03

*B. adolescentis *ATCC15703	*Bifidobacteriaceae*	4.10	732.95	3.95E-10
		
		2.90	338.59	5.59E-07

*L. ramnosus *DSM20021	*Lactobacillaceae, L. casei*	2.40	101.76	1.41E-03
		
		4.23	177.70	4.62E-07

*L. delbrueckii *DSM20074	*Lactobacillaceae*	3.77	210.11	2.24E-08
		
		3.10	121.93	6.27E-08

*L. pentosus *DSM20314	*Lactobacillaceae*	3.05	131.65	4.58E-09
		
		1.63	58.30	5.32E-07

*L. acidophilus *DSM20079	*Lactobacillaceae*	2.39	68.49	8.70E-05
		
		2.66	78.50	5.88E-06

*L. reuteri *DSM20016	*Lactobacillaceae*	3.17	150.57	4.66E-09
		
		1.74	83.60	1.98E-07

*L. plantarum *DSM21074	*Lactobacillaceae, L. plantarum*	2.12	197.32	3.79E-09
		
		2.09	148.35	2.77E-08

*C. difficile *ATCCBAA1382	*Clostridium *XI, *Clostridium difficile*	1.12	238.87	4.88E-04
		
		0.80	126.38	1.96E-03

*C. jejuni *ATCC33292	*Campylobacter jejuni*	0.70	19.89	5.29E-03
		
		0.91	28.44	5.69E-03

*V. parvula *ATCC10790	*Veillonella, Clostridium *IX	1.12	205.66	1.57E-04
		
		0.99	140.95	1.39E-04

*B. breve *DSM20091	*Bifidobacteriaceae*	2.22	570.01	6.22E-05
		
		1.69	289.07	2.72E-04

*B. longum *ATCC15707	*Bifidobacteriaceae, B. longum*	1.76	341.94	1.64E-03
		
		0.66	134.86	4.26E-02

*R. productus *ATCC 23340	*Clostridium *XIVa	0.64	4.21	1.41E-03
		
		1.06	17.16	1.24E-06

*L. salivarius *SV2	*Lactobacillaceae, L. salivarius*	0.89	12.23	4.34E-04
		
		0.65	7.27	2.69E-05

*E. faecalis *ATCC700802	*E. faecalis*	3.12	306.51	1.09E-03
		
		2.27	217.16	6.56E-03

*C. leptum *DSM73	*Ruminocuccos bromii Clostridium *IV	2.28	88.89	5.52E-07
		
		1.13	39.86	2.00E-07

*R. albus *DSM20455	*Ruminocuccos albus Clostridium *IV	1.46	47.05	2.50E-07
		
		1.41	32.01	4.37E-06

Table reporting the results of the tests to assess probe specificity: 28 bacterial DNA targets were chosen to validate the probe pairs. For each DNA analyzed we report: probe pair showing significant signals, SNR_s_, SNR_ns _(see main text for acronym definitions). The p-values of specific probes are reported for each duplicate experiment. Where needed (i.e. more than one probe pair was present), data are the average of the positive signals (for both SNR_s _and p-values)

#### Evaluation of the LDR sensitivity and relative abundance detection level

**Figure 2:**
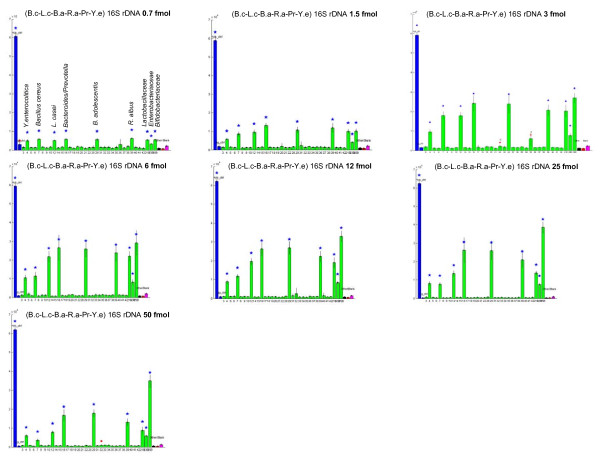
**Complex mix of 16 rRNA amplicons**. LDR-universal array experiments carried out on a complex mix of 16 rRNA amplicons obtained from six members of the human intestinal microbiota: *B. cereus*, *L. casei*, *B. adolescentis*, *R. albus*, *Prevotella*, *Y. enterocolitica*. Amplicons were tested in a concentration ranging from 0.7 to 75 fmol. Blue stars over the fluorescence bars indicate the probes that gave a positive response with a *P *< 0.01. Red dots indicate that one or two replicates out of four for each ZipCode were excluded because of having an IF < 2.5 times the average of the spots.

In order to define the detection limits of the HTF-Microbi.Array, LDR-UA experiments were carried out with different concentrations of an artificial mix of 16S rRNA amplicons from 6 members of the human intestinal microbiota. The 16S rRNA amplicons from *Bacillus cereus*, *Lactobacillus casei*, *Bifidobacterium adolescentis*, *Ruminococcus albus*, *Prevotella*, *Y. enterocolitica *were all specifically recognized in a range of concentrations from 0.7 to 75 fmol (*P *< 0.01), demonstrating the high sensitivity and specificity of the array (Fig. 2). Subsequently, in order to evaluate the relative abundance detection level of the HTF-Microbi.Array, LDR-UA experiments were performed on hybridization mixes containing low quantities of *Escherichia coli *PCR products and increasing amounts of human genomic DNA. This is a fundamental issue in the case of single species present in the gut microbiota at very low fractional abundance (< 0.1%) [21]. According to our data, 1 fmol of *E. coli *amplicon was sufficient (p < 0.005) to be detected in all the tested conditions (from up to 6.3 μg of human gDNA) (Additional file 4). Considering the PCR product as a ~1700 bp amplicon, 1 fmol corresponds to 1.2 ng and, thus, the sensitivity limit results 0.02%.

### Characterization of the faecal microbiota of eight healthy young adults

**Figure 3:**
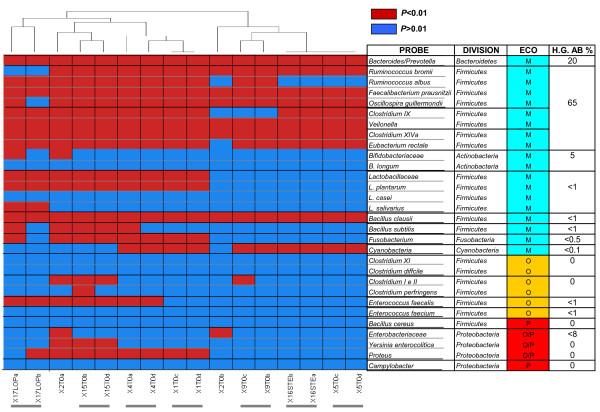
**Phylogenetic fingerprints**. Cluster analysis of the phylogenetic fingerprint of 16 faecal samples from 8 young adults. Response of each of the HTF-Microbi.Array probes for what concerns presence/absence of the target group is showed: positive response in red (P < 0.01), negative responses in blue (P > 0.01). Gary lines below the samples indicate adjacent replicated LDR of the same sample.

**Figure 4:**
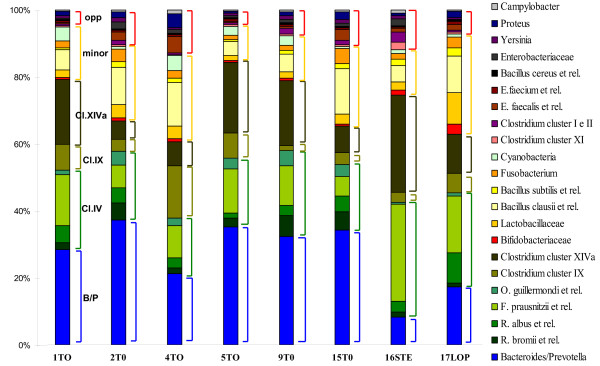
**IF relative contribution**. For each sample the entire HTF-Microbi.Array probe set was considered and their relative IF contribution was calculated as percentage of the total IF. Sub-probes were excluded and for each subject data from two separate LDR-universal array experiments were taken onto consideration. The averaged IF from both the LDR-Universal Array experiments was considered. The principal intestinal groups of major mutualistic symbionts are indicated: *Bacteroides/Prevotella *(B/P) blue, *Clostridium cluster *IV (Cl.IV) green, *Clostridium cluster *IX (Cl.IX) brown, *Clostridium cluster *XIVa (Cl.XIVa) dark brown. *Lactobacillus*, *B. clausii*, *B. subtilis*, *Fusobacterium *and *Cyanobacteria *are grouped as minor mutualistic symbionts (minor) indicated in yellow. *Proteus*, *Yersinia *and *E. faecalis *are grouped as opportunistic pathogens (opp) in red.

The HTF-Microbi.Array was applied in a pilot study for the characterization of the faecal microbiota of eight young adults. For all subjects faecal DNA was extracted, total bacterial 16S rRNA amplified, and two separate LDR-UA experiments were carried out (Additional file 5). For each sample a profile of presence-absence probes response was obtained. The cluster analysis of the phylogenetic fingerprints showed that, with the exception of subject n. 2, samples from the same subject clustered together. The reproducibility of the experiments was evaluated by considering the percentage of the probes giving the same response in both the technical replicates of each sample. With the exclusion of subject n. 2, an average reproducibility of 96% was obtained for all the subject under study, demonstrating a good reproducibility of the microbiota fingerprints obtained using the HTF-Microbi.Array (Fig. 3). As expected, the major mutualistic symbionts of the human intestinal microbiota, such as *Bacteroidetes *and the members of the *Clostridium *cluster IV and XIVa, were represented in the faecal microbiota of all the subjects. With the exception of *B. clausii et rel.*, minor mutualistic symbionts such as *Actinobacteria*, *Lactobacillaceae*, *B. subtilis et rel.*, *Fusobacterium*, and *Cyanobacteria *were detected only in different sub-fractions of the subjects. In particular, subjects n. 17, 15, 4, and 1 were characterized by the presence of *Fusobacterium*. Subjects n. 4, 15 and 17 possessed *B. subtilis et rel.*, while subjects n. 4, 1, 9, 16 and 5 harboured *Cyanobacteria *in their faecal microbiota. On the other hand, only a fraction of the subjects, clustering on the left side of the map, presented opportunistic pathogens in their faecal microbiota. Subjects n. 17, 15 and 4 presented both *Proteus *and *E. faecalis et rel.*, while in subject n. 15 members of the *Clostridium *cluster I and II and *Yersinia et rel. *were also detected. For each subject the relative fluorescence intensity (IF) contribution of each HTF-Microbi.Array probes, in terms of percentage of the total IF, was also calculated (Fig. 4). The mean of IF data from both the LDR-UA experiments were considered. Even if all subjects were characterized by a specific individual profile, a common trend can be found by comparing the comprehensive relative IF contribution of probes targeting major mutualistic symbionts (*Bacteroides/Prevotella*, *Clostridium *clusters IV, IX, and XIVa), minor mutualistic symbionts (*Bifidobacteriaceae, Lactobacillaceae*, *B. clausii et rel.*, *B. subtilis et rel.*, *Fusobacterium*, and *Cyanobacteria*), and opportunistic pathogens (*Clostridium *clusters I and II, IX, *E. faecalis et rel.*, *E. faecium et rel., B. cereus et rel., Enterobacteriaceae, Yersinia, Proteus, Campylobacter*). In particular, for all subjects the highest relative IF contributions were obtained for major mutualistic symbionts. The contribution of *Bacteroides/Prevotella *ranged between 8-37%, whereas the contribution of *Clostridium *clusters IV, IX, and XIVa ranged between 17-34%, 3-15%, and 5-29%, respectively. Differently, minor mutualistic symbionts were characterized by lower values of relative IF contributions. *Bifidobacteriaceae *contributed for the 0.5-3.1%, *Lactobacillaceae *for the 1.5-9.4%, *B. clausii et rel. *for the 4-13%, *B. subtilis et rel. *for the 0.6-2.5%, *Fusobacterium *for the 1.2-4.4%, and *Cyanobacterium *for 0.6-4.5%. As expected, opportunistic pathogens showed together the lowest relative IF contribution in all the subjects under study (from 5 to 10%).

## Discussion

In these last years, 16S rRNA microarrays emerged as a sensitive and efficient way to screen complex bacterial communities. Here we describe and validate the HTF-Microbi.Array, a new phylogenetic DNA microarray designed for the high taxonomic level fingerprint of the human intestinal microbial community. The HTF-Microbi.Array is based on the LDR-UA approach, which is a fast and sensitive tool for the characterization of complex microbial communities with high sensitivity and specificity [25,26]. The use of this molecular technique allows overcoming the major limitations of DNA microarrays whose discriminative power is based on hybridization. In fact, a) optimization of the hybridization conditions for each probe set is not required; b) problems due to the secondary structures of the target DNA are minimized, c) steric hindrances of differentially sized nucleic acid hybrids formed on the array after the hybridization are decreased [29]. The final probe set of the HTF-Microbi.Array allows a high taxonomic level fingerprint of the human intestinal microbiota, with a good coverage of the major and minor components, as well as some of the most important pathogens and opportunistic bacteria [30]. The LDR probes were designed by choosing DS oligonucleotides whose 3'end allowed the perfect discrimination of the target species from the non-target ones on the basis of our 16S rRNA sequence database. Definition of accurate and specific negative sets of gut microbiota sequences by ORMA tool [31] allowed the selection of maximally discriminative probe pairs. Probe specificity was confirmed on the entire known 16S rRNA gene sequences environment by the RDP Probe Match tool. This requirement is fundamental, since the primer set used for the PCR amplification was the "universal" 16S rRNA primer set designed by Edwards and co-workers [32].

The HTF-Microbi.Array recognized without ambiguity the 16S rRNA amplicons obtained from 28 members of the intestinal microbiota belonging to *Bacteroides/Prevotella*, *Clostridium *clusters IV, IX, XIVa, XI, I and II, *Bifidobacteriaceae*, *Lactobacillaceae*, *Bacillus*, *Enterococcus*, *Enterobacteriaceae *and *Campylobacter*, demonstrating the specificity of all the probe pairs. The sensitivity of the HTF-Microbi.Array was evaluated by using different concentrations of an artificial mix of 16S rRNA amplicons obtained from 6 microorganisms members of the human intestinal microbiota. To compensate the eventual drop in the signal due to a very low target concentrations, lower than 0.7 fmol (i.e. a percentage lower than 1.5% of the commonly used quantity of 50 fmol), a slightly relaxed criteria for significance of the t-test to α = 0.05 was chosen. All PCR products were specifically recognized in a concentration range from 75 to 0.7 fmol, showing high array sensitivity. The efficiency of the HTF-Microbi.Array in the detection of a particular target in a complex DNA environment was also determined. According to our data, the array is able to detect a specific DNA target down to 0.02% of the total 16S rRNA, which is comparable to the values obtained by Rajilic-Stojanovic *et al. *[23] and Palmer *et al. *[21]. Thus the HTF-Microbi.Array shows the potentiality to sense low abundant species of the gastrointestinal microbiota, enabling the detection of the 16S rRNA of a peculiar target group present at a fractional abundance <0.1% in an artificial mixture.

The HTF-Microbi.Array was used in a pilot study to characterize the faecal microbiota of eight young adults. Faecal microbiota was chosen as DNA source since sample collection is not invasive, samples contain large amount of microbes, and, most important, it is representative of interpersonal differences in distal gut microbial ecology [33]. In order to have a good representation of the less abundant species of the intestinal microbial community, LDR reactions were performed starting from 50 fmol of PCR product. Cluster analysis of the presence-absence probes profiles enabled the identification of a reproducible high taxonomic level microbiota fingerprint for each subject. As expected, the intestinal microbial community of the voluntaries in the study resembled the typical fingerprint of healthy adults [28]. According to our data, the faecal microbiota of the enrolled subjects was dominated by major mutualistic symbionts. In fact, members of *Bacteroidetes, Clostridium *clusters IV, IX and XIVa were all represented in 100% of the subjects. On the other hand, minor mutualistic symbionts, such as *Lactobacillaceae*, *B. subtilis et re.*, *Fusobacterium *and *Cyanobacteria*, were detected in 55, 37, 50, and 63% of the subjects, respectively. Opportunistic pathogens, such as *E. faecalis et rel.*, members of the *Clostridium *cluster I and II and *Enterobacteriaceae*, were represented only in 43, 25 and 12% of the subjects, respectively. Most importantly, enteropathogens such as, *C. difficile*, *C. perfringens*, *E. faecium et rel.*, *B. cereus et rel.*, and *Campylobacter *were never detected. A discrepancy between our data and the literature is the relatively low prevalence of the health promoting *Bifidobacteriaceae *in our samples (only 13% of samples). However, the low prevalence of bifidobacteria is a typical bias for several phylogenetic DNA microarrays [22,23]. Probably this is due to the intrinsic low efficiency of amplification of the bifidobacterial genome with universal primer sets for the 16S rRNA gene [8]. Surprisingly, a high prevalence was obtained for the minor mutualistic symbiont *B. clausii et rel.*, 100% of samples, and the opportunistic pathogen *Proteus*, 50% of samples. For each subject the relative IF contributions of the probes were calculated, obtaining an approximate evaluation of the relative abundance of the principal microbial groups of the faecal microbiota. In general agreement with previous metagenomic studies [7-11] and SSU rRNA phylogenetic microarray investigations [22,23], mutualistic symbionts such as *Bacteroidetes, Clostridium *clusters IV, IX and XIVa largely dominated the faecal microbiota, contributing for the 65 to 80% of total microbiota, depending on the subject. Differently, with an overall contribution ranging from 10 to 30%, minor mutualistic symbionts such as *B. clausii et rel., Bifidobacteriaceae*, *Lactobacillaceae*, *B. subtilis et rel.*, *Fusobacterium*, and *Cyanobacteria *were largely subdominant. Opportunistic pathogens represented only a small fraction of the intestinal microbiota. Even if subjects under study show a common trend when the ratio between the relative IF of major, minor and opportunistic components were considered, differences in the relative IF contribution of single probes were detectable and subject specific profiles were identified. For instance, subject n. 1 showed a higher relative fluorescence for probes targeting major mutualistic symbionts and a lower relative fluorescence for minor mutualistic symbionts and opportunistic pathogens than subjects n. 4 and 15. On the other hand subjects n. 15 and 17 were characterized by a lower ratio *Bacteroidetes*/*Firmicutes *with respect to all the other subjects. It is tempting to hypothesize that differences in relative IF contribution within samples could represent an approximation of differences in relative abundances of the targeted groups in the faecal microbiota. However, caution must be taken when microarray based methods for the relative quantification of bacterial groups in complex microbial communities are used. In fact, biases are introduced at several levels of the experimental procedure: DNA extraction and purification, PCR amplification of the 16S rRNA gene, and interspecies variation of the rRNA gene copy number [21].

## Conclusion

The HTF-Microbi.Array has been revealed a fast and sensitive tool for the high taxonomic level fingerprint of the human intestinal microbiota in terms of presence/absence of the principal groups. Since the flexibility of the universal array platform allow the addition of new probe pairs without a further optimization of the hybridization conditions [25,26], the HTF-Microbi.Array can be easy implemented with the addition of new probe pairs targeting emerging microbial groups of the human intestinal microbiota, such as, for instance, the mucin degrading bacterium *Akkermansia muciniphila *[34]. The evaluation of the relative abundance of the target groups on the bases of the relative IF probes response still has some hindrances. However, considered all the possible biases (i.e. DNA extraction/purification, PCR, copy number variations, etc.) typical of the microarray technology, analysis of IFs from our LDR-UA platform can be useful in the estimation of the relative abundance of the targets groups within each sample. Focusing the phylogenetic resolution at division, order and cluster levels, the HTF-Microbi.Array results blind with respect to the inter-individual variability at the species level. Its potential to characterize the high order taxonomic unbalances of the human intestinal microbiota associated with specific diseases will be assessed in further studies.

## Methods

### Recruitment

Eight healthy Italian individuals of 30 years old were enrolled for the study. None of the subjects had dietary restrictions except for antibiotics, probiotics and functional foods for at least 4 weeks prior to sampling. None of the selected subjects had a history of gastrointestinal disorders at the time of sampling. The study protocol was approved by the Ethical committee of Sant'Orsola-Malpighi Hospital (Bologna, Italy) and an informed consent was obtained from each enrolled subject. Faeces were collected for each subject and stored at -20°C.

### Bacterial strains and culture conditions

The bifidobacterial strains used in this study were *Bifidobacterium adolescentis *ATCC15703, *B. bifidum *DSM20456, *B. breve *DSM20091, *B. longum *ATCC15707. The *Lactobacillus *strains were *Lactobacillus plantarum *DSM21074, *L. casei *DSM20011, *L. ramnosus *DSM20021, *L. salivarius *SV2 (strain from our collection), *L. delbrueckii *DSM 20314, *L. gasseri *DSM20243, *L. reuteri *DSM20016, *L. pentosus *DSM20134, *L. acidophilus *DSM20079. All bifidobacteria and *Lactobacillus *strains were grown on De Man-Rogosa-Sharpe (MRS) broth with cysteine (0.5 g/l) at 37°C under an anaerobic atmosphere (Anaerocult, Merck, Darmstadt, Germany). *Escherichia coli *ATCC11105 was cultivated at 37°C aerobically on TY-broth. *Salmonella cholerasuis typhimurium *and *Yersinia enterocolitica*-type, kindly provided by A. Essig, Dept. of Medical Microbiology, University of Ulm, Germany, were cultivated aerobically at 30°C on BHI-broth.

### Target selection and consensus extraction

A database of 16S rRNA sequences was created by integration of the 16S rRNA database of the ARB Project (release February, 2005) (http://www.arb-home.de; [35]) with the database of the Ribosomal Database Project (RDP; release September, 2007) (http://rdp.cme.msu.edu/; [36,37]). A phylogenetic tree was obtained in the ARB software, by using the neighbour-joining algorithm for the sequence alignment. The tree was used for the rational selection of phylogenetically related groups of bacteria belonging to the human intestinal microbiota which correspond to nodes of the phylogenetic tree (Additional file 1). Group specific consensus sequences were extracted, with a cut-off of 75% for base calling. Nucleotides which occurred at lower frequencies were replaced by the appropriate IUPAC ambiguity code.

### Probe design

Multiple alignment step of the selected sequences was performed in ClustalW [38]. Since the taxonomic classification of the 30 groups selected for the probe design varied from species to phylum level, careful grouping of the sequences was performed for the multiple alignment step: (a) for higher level probes, only family/phylum consensus sequences were used as a negative set for probe design; (b) for genus/species level probes, only sequences belonging to other families/phyla were selected. All the LDR probe pairs were designed using ORMA [31]. Both DS and CP were required to be between 25 and 60 bases pair, with a Tm of 68 ± 1°C, and with maximum 4 degenerated bases. *In-silico *check versus a publicly available database (i.e.: RDP) was then performed for assessing probe pair specificity.

### DNA extraction

Total DNA was extracted from 10^9 ^bacterial cells by using the DNeasy Tissue Kit 50 (Quiagen, Düsseldorf, Germany) following the manufacturer instructions. Bacterial DNA was also extracted from lyophilized bacterial cells of the following DSMZ (Braunschweig, Germany) collection strains: *Clostridium leptum *DSM73, *Ruminococcus albus *DSM20455, *Eubacterium siraeum *DSM15700, *C. viride *DSM6836, *Megasphera micrinuciformis *DSM17226, *Bacillus clausii *DSM2515, *B. subtilis *DSM704, *B. cereus *DSM21, and *Proteus mirabilis *DSM4479. Lyophilized bacterial cells were suspended in 1 ml of lysis buffer (500 mM NaCl, 50 mM Tris-HCl pH 8, 50 mM EDTA, 4% SDS) and DNA extraction was carried out by employing the same procedure used for the extraction of genomic DNA from faecal samples, according to the following procedure. Total DNA from faecal material was extracted using QIAamp DNA Stool Min Kit (Qiagen) with a modified protocol. 250 mg of faeces were suspended in 1 ml of lysis buffer. Four 3 mm glass beads and 0.5 g of 0.1 mm zirconia beads were added, and the samples were treated in FastPrep (MP Biomedical, Irvine, CA, USA) at 5.5 ms for 3 min. Samples were heated at 95°C for 15 minutes, then centrifuged for 5 min at full speed to pellet stool particles. Supernatants were collected and 260 μl of 10 M ammonium acetate were added, followed by incubation in ice for 5 min and centrifugation at full speed for 10 min. One volume of isopropanol was added to each supernatant and incubated in ice for 30 min. The precipitated nucleic acids were collected by centrifugation for 15 min at full speed and washed with 70% ethanol. Pellets were resuspended in 100 μl of TE buffer and treated with 2 μl of DNase-free RNase (10 mg/ml) at 37°C for 15 min. Protein removal by Proteinase K treatment and DNA purification with QIAamp Mini Spin columns were performed following the kit protocol. 200 μl of TE buffer were used for DNA elution. Final DNA concentration was determined by using NanoDrop ND-1000 (NanoDrop Technologies, Wilmington, DE). The bacterial DNA from the following 11 ATCC strains was directly obtained from the ATCC: *Bacteroides fragilis *ATCC25285, *B. thetaiotaomicron *ATCC29148, *Prevotella melaninogenica *ATCC25845, *Veilonella parvula *ATCC10790, *C. difficile *ATCCBAA1382, *C. acetobutilicum *ATCC824, *C. perfringens *ATCC13124, *Enterococcus faecalis *ATCC700802, *E. faecium *ATCC51559, *Campylobacter jejuni *ATCC33292, *R. productus *23340.

### Polymerase Chain Reaction (PCR)

All the oligonucleotide primers and probe pairs were synthesized by Thermo Electron (Ulm, Germany). PCR amplifications were performed with Biometra Thermal Cycler II and Biometra Thermal Cycler T Gradient (Biometra, Germany). PCR products were purified by using a Wizard SV gel and PCR clean-up System purification kit (Promega Italia, Milan, Italy), according to the manufacturer's instructions, eluted in 20 μl of sterile water, and quantified with the DNA 7500 LabChip Assay kit and BioAnalyzer 2100 (Agilent Technologies, Palo Alto, CA, USA). 16S rRNA was amplified using universal forward primer 16S27F (5'-AGAGTTTGATCMTGGCTCAG-3') and reverse primer r1492 (5'-TACGGYTACCTTGTTACGACTT-3'), following the protocol described in Castiglioni *et al. *[25] except for using 50 ng of starting DNA and 0.5 U of DNAzyme DNA polymerase II (Finnzymes, Espoo, Finland).

### LDR/Universal Array approach

Phenylen-diisothiocyanate (PDITC) activated chitosan glass slides were used as surfaces for the preparation of universal arrays [39], comprising a total of 49 Zip-codes. Hybridization controls (cZip 66 oligonucleotide, complementary to zip 66, 5'-Cy3-GTTACCGCTGGTGCTGCCGCCGGTA-3') were used to locate the submatrixes during the scanning. The entire experimental procedure for both the chemical treatment and the spotting is described in detail in Consolandi *et al. *[40]. An overview of the Universal Array layout and ZipCodes is provided as Additional file 6. Ligase Detection Reaction and hybridization of the products on the universal arrays were performed according to the protocol described in Castiglioni *et al. *[25], except for the probe annealing temperature, set at 60°C.

The LDRs were carried out in a final volume of 20 μl with different quantities of purified PCR products: a) all LDRs for specificity tests were performed on 50 fmol of initial PCR product, for having no issues related to target; b) sensitivity tests were performed with decreasing PCR product concentration from 75 to 0.7 fmol; c) relative abundance tests were performed on 1 fmol *E. coli *PCR amplicon, mixed with human genomic DNA extracted from whole blood, at decreasing concentrations, from 4%, down to 0.02%; d) LDR experiments on the eight faecal samples were performed on 50 fmol of PCR product.

### Data analysis

All arrays were scanned with ScanArray 5000 scanner (Perkin Elmer Life Sciences, Boston, MA, USA), at 10 μm resolution. In the experiments, the fluorescent images were obtained with different acquisition parameters on both laser power and photo-multiplier gain, in order to avoid saturation. IF were quantitated by ScanArray Express 3.0 software, using the "Adaptive circle" option, letting diameters vary from 60 to 300 μm. No normalization procedures on the IFs have been performed. To assess whether a probe pair was significantly above the background (i.e. was "present" or not), we performed a one-sided t-test (α = 0.01). The criteria was relaxed to α = 0.05 for sensitivity tests. The null distribution was set as the population of "Blank" spots (e.g. with no oligonucleotide spotted, n = 6). Two times the standard deviation of pixel intensities of the same spots was added to obtain a conservative estimate. For each zip-code, we considered the population of the IFs of all the replicates (n = 4) and tested it for being significantly above the null-distribution (H_0_: μ_test _= μ_null_; H_1_: μ_test_>μ_null_). In case one replicate in the test population was below 2.5 times the distribution mean, this was considered an outlier and was discarded from the analyses. We calculated the ratio between the signal intensities of the specific probes on the blank intensity (SNR_s_) and the ratio between all the other probes and the blank intensity (SNR_ns_).

### Clustering

Hierarchical clustering of HTF-Microbi.Array profiles was carried out using the statistical software R http://www.r-project.org. The Euclidean distance among sample profiles was calculated and Ward's method was used for agglomeration.

## Authors' contributions

MC, CC, MS, and EB performed the study design, analysis and interpretation of the data and the writing of the paper. BC and BV participated in the design of the study. GDB and PB coordinated the study. All authors read and approved the manuscript.

## Supplementary Material

Additional file 1 **HTF-Microbi.Array target groups**. Phylogenetically related groups target of the HTF-Microbi.Array. Click here for file

Additional file 2 **HTF-Microbi.Array probe list**. Table of the 30 designed probe pairs. Sequences (5' -> 3') for both DS and CP are reported, as well as major thermodynamic parameters (melting temperature, length, number of degenerated bases). Click here for file

Additional file 3 **Specificity tests of the HTF-Microbi.Array**. Raw data of the specificity tests of the HTF-Microbi.Array. Each column represent a different sample, whose identification is reported as its label. On the left, the ZipCode, the probe name and ID are reported. "Type" is a numeric flag used for the classification of the probes: 1 is the hybridization control, 2 is the ligation control, 3 indicates the HTF-Microbi.Array probes, 4 are the unused ZipCodes and 5 is the Blank. "Numeric ID" is given to the probes according to their "type" and "Oligo ID" values. Click here for file

Additional file 4 **Sensitivity tests of the HTF-Microbi.Array**. Raw data of the sensitivity tests on the HTF-Microbi.Array. The workbook has two spreadsheets: "Artificial mix data", reporting the results of the serial dilutions of the 6 bacterial DNA mix (*B. cereus*, *L. casei*, *B. adolescentis*, *R. albus*, *Prevotella*, *Y. enterocolitica*), with concentrations ranging from 50 to 0.7 fmol. "Absolute sensitivity *E. coli*" spreadsheet reports the results of the tests on low quantities of *E. coli *16S amplicon in increasing amounts of human genomic DNA. The file is structured as described above for Additional file 3. Click here for file

Additional file 5 **Tests of the HTF-Microbi.Array on faecal samples**. Raw data for the experimental characterization of the faecal microbiota of eight healthy young adults. Patient ID and replicate number are reported as the column headers. The file is structured as described above for Additional file 3. Click here for file

Additional file 6 **Universal array scheme**. Graphical representation of the Universal Array platform. Each array has 8 identical subarrays (A), which can be addressed independently. Each subarray is made by 208 spots, with quadruplicates of each ZipCode (B); hybridization and ligation controls and Blanks are repeated 8, 6 and 6 times, respectively; the figure highlights in gray the ZipCodes actually associated to probe pairs used in the HTF-Microbi.Array. Sequences (5' - 3' oriented) and numbers of the ZipCodes are reported in (C). Click here for file
