# Supplementary material for: High taxonomic level fingerprint of the human intestinal microbiota by Ligase Detection Reaction - Universal Array approach
Source: BMC Microbiol. 2010 Apr 19;10:116. doi: 10.1186/1471-2180-10-116 (PMC2873488; doi:10.1186/1471-2180-10-116)
Supplement: Additional file 6 — Universal array scheme. Graphical representation of the Universal Array platform. Each array has 8 identical subarrays (A), which can be addressed independently. Each subarray is made by 208 spots, with quadruplicates of each ZipCode (B); hybridization and ligation controls and Blanks are repeated 8, 6 and 6 times, respectively; the figure highlights in gray the ZipCodes actually associated to probe pairs used in the HTF-Microbi.Array. Sequences (5' - 3' oriented) and numbers of the ZipCodes are reported in (C). [file 1471-2180-10-116-S6.PDF]

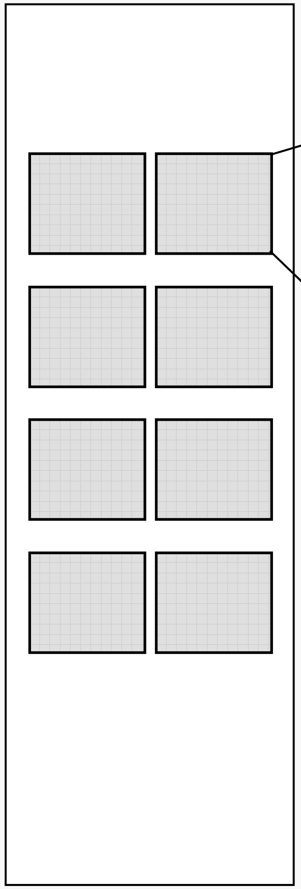

(a)

**66**	63	**Blank**	1	2	3	4	5	1	2	3	4	5	**Blank**	63	**66**
6	7	8	9	10	12	14	15	6	7	8	9	10	12	14	15
16	17	18	19	20	21	22	23	16	17	18	19	20	21	22	23
24	27	28	29	31	32	33	34	24	27	28	29	31	32	33	34
35	36	37	38	39	40	41	42	35	36	37	38	39	40	41	42
44	1B	3B	5B	11B	13B	15B	21B	44	1B	3B	5B	11B	13B	15B	21B
**66**	63	**Blank**	1	2	3	23B	25B	23B	25B	1	2	3	**Blank**	63	**66**
4	5	6	7	8	9	10	12	4	5	6	7	8	9	10	12
14	15	16	17	18	19	20	21	14	15	16	17	18	19	20	21
22	23	24	27	28	29	31	32	22	23	24	27	28	29	31	32
33	34	35	36	37	38	39	40	33	34	35	36	37	38	39	40
41	42	44	1B	3B	5B	11B	13B	41	42	44	1B	3B	5B	11B	13B
**66**	63	**Blank**	66	15B	21B	23B	25B	15B	21B	23B	25B	66	**Blank**	63	**66**

(b)

ZipCode Number	Sequence 5' -> 3'
1	GATGATCGACGAGACACTCTCGCCA
2	CGGTCGACGAGCTGCCGCGCAAGAT
3	GACATTCGCGATCGCCGCCCGCTTT
4	CGGTATCGCGACCGCATCCCAATCT
5	GCTCGAAGAGGCGCTACAGATCCTC
6	CACCGCCAGCTCGGCTTCGAGTTCG
7	CGACTCCCTGTTTGTGATGGACCAC
8	CTTTTCCCGTCCGTCATCGCTCAAG
9	GGCTGGGTCTACAGATCCCCAACTT
10	GAACCTTTCGCTTCACCGGCCGATC
12	TTTCGGCACGCGCGGGATCACCATC
14	CTCGGTGGTGCTGACGGTGCAATCC
15	TCAACGTGCCAGCGCCGTCCTGGGA
16	GCGAAGGAACTCGACGTGGACGCCG
17	CGGGGATACCGATCTCGGGCGCACA
18	GGAGCTTACGCCATCACGATGCGAT
19	CGTGGCGGTGCGGAGTTTCCCCGAA
20	CGATCCAACGCACTGGCCAAACCTA
21	CTGAATCCTCCAACCGGGTTGTCGA
22	TTCGGCGCTGGCGTAAAGCTTTTGG
23	GTAAATCTCCAGCGGAAGGGTACGG
24	CCGGCTTTGAACTGCTCACCGATCT
27	ACTACGCAACACCGAACGGATACCC
28	GGACCAATGGTCCCATTGACCAGGT
29	CAACGCTGAGCGCGTCACTGACATA
31	GAGACAAAGGTCTGCGCCAGCACCA
32	TGGCCACACTGTCCATTTGCGCGGT
33	CCTTGCGACGTGTCAAGTTGGGGTC
34	AGGTTAGGGTCGCGCCAAACTCTCC
35	ACGACTGCGAGGTGCGGTAAGCACA
36	GCGATCGCCGGGAGATATACCCAAC
37	TCGTGCCGGACTCGAGCACCAATAC
38	GCTTTAGCACCGCGATGGCGTAGAC
39	CAGCCGCGGTACTGAATGCGATGCT
40	CCCCGGATAGCTGACGAGGCTTACG
41	TCCGGACAGGTTGGGGTGCGTTTGG
42	CGTAGAGCAACGCGATACCCCCGAC
44	AGCAGCAGTGACAATGCCACCGCCG
1B	TGCGACCTCAGCATCGACCTCAGC
3B	CAGCACCTGACCATCGATCGCAGC
5B	GACCACCTTGCGATCGGGTACAGC
11B	TGCGGGTACAGCACCTACCTTGCG
13B	CAGCGGTAGACCACCTATCGTGCG
15B	GACCGGTATGCGACCTGGTATGCG
21B	TGCGATCGCAGCGGTAACCTGACC
23B	CAGCATCGGACCGGTAATCGGACC
25B	GACCATCGTGCGGGTAGGTAGACC
63	TGGCACCGACGGCTGGCACACCAC
66	TACCGGCGGCAGCACCAGCGGTAAC

(c)
